# Prevalence of malocclusion in early childhood and its associated factors in a primary care service in Brazil

**DOI:** 10.1590/2317-1782/20212021007

**Published:** 2021-11-22

**Authors:** Natalia de Abreu Pegoraro, Camila Mello dos Santos, Beatriz Carriconde Colvara, Rafaela Soares Rech, Daniel Demétrio Faustino-Silva, Fernando Neves Hugo, Juliana Balbinot Hilgert

**Affiliations:** 1 Universidade Federal do Rio Grande do Sul – UFRGS - Porto Alegre (RS), Brasil; 2 Programa de Pós-Graduação em Avaliação de Tecnologias para o SUS, Grupo Hospitalar Conceição – GHC - Porto Alegre (RS), Brasil; 3 Universidade Federal de Ciências da Saúde de Porto Alegre – UFCSPA - Porto Alegre (RS), Brasil

**Keywords:** Malocclusion, Primary Health Care, Pediatric Dentistry, Breast Feeding, Pacifiers, Preventive Dentistry, Má Oclusão, Atenção Primária à Saúde, Odontopediatria, Amamentação, Chupeta, Odontologia Preventiva

## Abstract

**Purpose:**

to evaluate the prevalence of malocclusion and its associated factors of children cared for by a PHC Service in Porto Alegre, Brazil.

**Methods:**

a cross-sectional study nested in a cohort, carried out in 12 Health Care Practices. Of the 414 children in the cohort examined, 268 were assessed for malocclusion. The presence of anterior open bite, posterior and anterior crossbite was evaluated by the criteria of Foster and Hamilton. Socioeconomic variables, breastfeeding habits and pacifier use information were collected through a standardized questionnaire. Data analysis was performed using a hierarchical approach by Poisson Regression with robust variance.

**Results:**

out of the total 268 evaluated, 135 (50.4%) were boys, and the average age was 28.6 (± 11.9) months. Out of the 143 (53.4%) cases of malocclusion, 113 were anterior open bite, 16 were anterior crossbite, 27 were posterior crossbite, and 38 had increased overjet. In the final analysis, it was observed that there was a higher prevalence of malocclusion in children who never breastfed (PR = 1.44; 95%CI 1.00-2.08) and who always used a pacifier to sleep (PR = 1.81; 95%CI 1.14-2.86).

**Conclusion:**

the prevalence of malocclusion in this population was high and was associated with behavioral habits, such as the use of pacifier and not breastfeeding.

## RESUMO

**Objetivo:**

avaliar a prevalência de maloclusões e seus fatores associados em crianças atendidas em um Serviço de APS de Porto Alegre, Brasil.

**Método:**

estudo transversal aninhado a uma coorte, realizado em 12 Unidades de Saúde. Das 414 crianças da coorte examinadas, 268 foram examinadas para maloclusão. A presença de mordida aberta anterior, mordida cruzada posterior e anterior foi avaliada pelos critérios de Foster e Hamilton. Variáveis socioeconômicas, hábitos de amamentação e informações sobre o uso de chupeta foram coletados por meio de questionário padronizado. A análise dos dados foi realizada por meio de uma abordagem hierárquica por Regressão de Poisson com variância robusta.

**Resultados:**

do total de 268 avaliados, 135 (50,4%) eram meninos e a média de idade foi de 28,6 (± 11,9) meses. Dos 143 (53,4%) casos de maloclusão, 113 eram mordida aberta anterior, 16 eram mordida cruzada anterior, 27 eram mordida cruzada posterior e 38 tinham overjet acentuado. Na análise final, observou-se que houve maior prevalência de maloclusões em crianças que nunca foram amamentadas (RP = 1,44; IC95% 1,00-2,08) e que sempre usaram chupeta para dormir (RP = 1,81; IC95% 1,14 -2,86).

**Conclusão:**

a prevalência de maloclusões nesta população foi elevada e esteve associada a hábitos comportamentais, como o uso de chupeta e a não amamentação.

## INTRODUCTION

Malocclusion is any change in the normal pattern of occlusion and dental arches, and its etiology is caused by hereditary and environmental factors, such as the incorporation of harmful oral habits, such as finger and pacifier sucking^(1,2)^. These changes interfere with the balance of the stomatognathic system and the children and adolescents’ quality of life^(3-5)^, reflecting on the comfort of eating, sleeping and engaging in social interaction, their self-esteem and their satisfaction with their oral health.

Malocclusion is the third most prevalent oral disorder in the Brazilian population. Approximately 66.7% of the children at the age of 5 have at least one type of malocclusion. At the age of 12, 37.7% have some type of malocclusion, with 17.7% of these being classified as severe or extremely severe^(6)^. The prevalence of malocclusion in the population of preschoolers is around 64.5%, and of these, 38.6% have more than one type of malocclusion simultaneously^(7,8)^.

A protective factor against malocclusion is breastfeeding, as it increases the possibility of adequate dental occlusion by strengthening the orofacial muscles^(9)^. The World Health Organization (WHO) recommends exclusive breastfeeding in the first six months of the baby's life, and the literature shows that the presence of malocclusion is approximately 60% higher in children who have never breastfed or breastfed for less than six months^(10)^.

Studies indicate that between 55% and 77% of children have at least one sucking habit^(11,12)^, and there is an association between the habit of sucking a pacifier and the presence of posterior crossbite in 36% of children^(13)^. Due to the high prevalence of malocclusion in the population, it is important to implement preventive measures to prevent the incorporation of harmful oral habits, encouraging guidance on the consequences that these habits can generate and the importance of early interception. There is evidence that, at an early stage, the orthodontic care is less complex and less costly ^(14)^. It is in this context that Primary Health Care (PHC) becomes essential, as it can provide care to more than 80% of health needs, in addition to uniting prevention and health promotion actions^(15)^. Although preventive guidelines for child malocclusions are included in the National Oral Health Policy of the Ministry of Health of Brazil, there seems to be difficulties for oral health teams in implementing effective actions for this disease. In this sense, knowing the aspects related to malocclusion in early childhood and in the context of PHC can contribute to the establishment of early preventive programs. The objective of the present study was to evaluate the prevalence of malocclusion and its associated factors in the early childhood of children cared for by a PHC Service in Porto Alegre, Rio Grande do Sul, Brazil.

## METHODS

This is a cross-sectional study nested in a cohort study carried out in the 12 Health Care Practices (HCPs) of the Community Health Service (SSC) of Grupo Hospitalar Conceição (GHC), in the city of Porto Alegre, Rio Grande do Sul, Brazil^(16)^.

**Figure 1:**
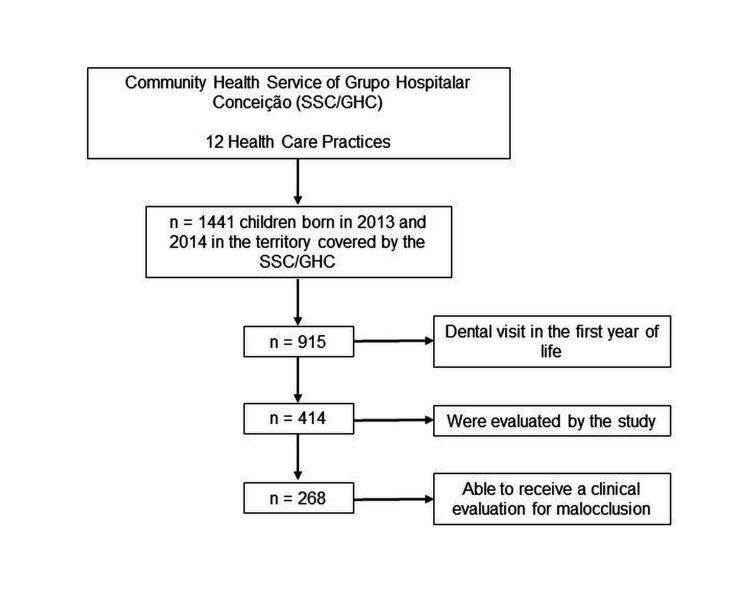
Study flowchart.

The universe of the cohort is of children born between 2013 and 2014, in the area covered by the SSC, which is composed of 1,441 children. Of these, 414 were evaluated by the study, but only 268 children were able to receive a clinical evaluation for malocclusion, as only those with erupted molars were evaluated for these conditions (Figure 1). Children and mothers/primary caregivers with a medical diagnosis of physical or cognitive disabilities that resulted in a lack of conditions to answer the questionnaires were excluded, as well as children outside the territory covered by the HCP for six consecutive months or more.

All mothers were invited to attend their respective HCP or to receive home visits from the examiners, who were always accompanied by Community Health Agents (CHA). All participants signed two copies of the Free and Informed Consent Form and received explanations about how the research works. The children were examined in the dental office, seated, with directed reflector light and the aid of wooden spatulas or odontoscope to move soft tissues away. During home visits, the child was seated in a chair, headlights made the intra-oral lighting, and wooden spatulas and odontoscope were also available. Besides the clinical examination, socioeconomic variables, breastfeeding habits and pacifier use information were collected through a standardized questionnaire.

The clinical examination for malocclusion was performed based on the criteria of Foster and Hamilton^(17)^, which assesses the presence of anterior open bite, posterior crossbite (unilateral or bilateral), adequate lip sealing, and overjet. The measurement for overjet was performed with a millimeter-periodontal probe at the most significant distance between the incisal edges of the upper and lower incisors. The overjet was classified as normal or accentuated (greater than or equal to 3 mm). Sixteen examiners received calibration by the *in lux* method^(18,19)^ for malocclusion, in which each examiner individually evaluated photos of 20 children that contained one of the occlusal changes under study, repeating the process after seven days. The respective mean intra- and interexaminer weighted kappa values were 0.88 (minimum = 0.77) and 0.83 (minimum = 0.74). The examiner applied the questionnaires, read in full, and the interpretation was at individual discretion.

**Figure 2:**
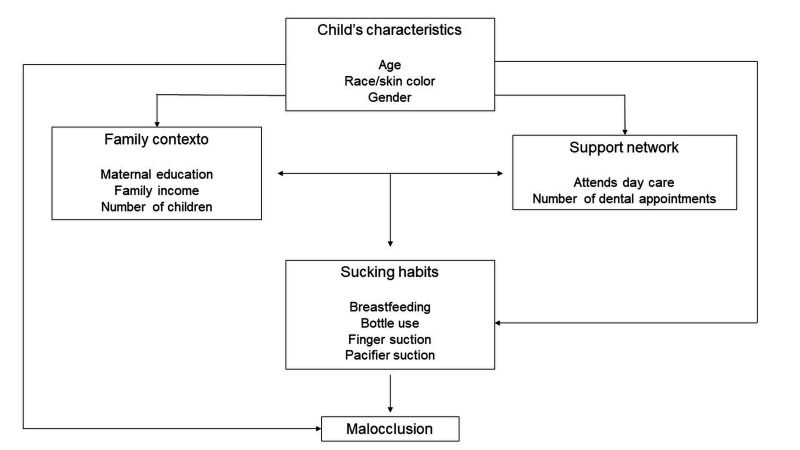
Block and hierarchical theoretical model for investigating factors associated with malocclusion.

Absolute and relative frequencies were calculated, chi-square test or Fisher's exact test were used to assess the associations between the variables studied and malocclusion, maintaining a significance level of 5%. Mann-Whitney test was performed for the quantitative variable that did not present a normal distribution. Data analysis was performed using a hierarchical approach (Figure 2), with the model divided into four blocks: 1) variables of child's characteristics (age, race/skin color, sex); 2) variables related to the family context (maternal education, family income, number of children), 3) support network (attends daycare, dental consultation); and 4) sucking habits (breastfeeding, bottle use, sucking finger, and pacifier use). The hierarchical approach used univariate Poisson regression models to estimate the relationships between the variables studied and the outcome. Multivariate Poisson regressions were also performed within each block. The presence of multicollinearity was evaluated by means of the variance inflation factor (VIF) estimates. The statistical significance of the prevalence ratio indices was assessed using the Wald test. All data were analyzed using SPSS v18 software.

The sample power was used to analyze associated factors, and the parameters used were the confidence level of 95%, the prevalence of the outcome and the associated factors found in this sample, estimating a minimum power of 78%.

The project was approved by GHC’s Ethics Committee (protocol number 13-063 and CAAE number 15015013.0.0000.5530).

## RESULTS

The clinical examination was performed on 268 children. The total prevalence of malocclusion was 143 (53.4%), and there were 113 (41.1%) cases of anterior open bite, 16 (5.9%) cases of anterior crossbite, 27 cases of posterior crossbite (10.0%), and 38 cases of increased overjet (14.5%). The average age of children at the time of the clinical examination was 28.6 (± 11.9) months.

The relationship between identification characteristics (block 1), socioeconomic characteristics (block 2), children's activities (block 3), and breastfeeding practices (block 4) with the presence of malocclusion is described in Table 1. Crude and adjusted Prevalence Ratios are described in Table 2. After analyzing the individual blocks, none of the variables in blocks 1 remained in the adjustment with the other blocks. In the final analysis, it is observed that there was a higher prevalence of malocclusion in children who never breastfed (PR = 1.44; 95%CI 1.00-2.08) and who always used a pacifier to sleep (PR = 1.81; 95%CI 1.14-2.86).

**Table 1:** Relationship between identification (block 1), socioeconomic (block 2), child's activities (block 3), and breastfeeding (block 4) characteristics with the presence of malocclusion. Porto Alegre, RS

	**Malocclusion**		
**Variable**	Absence n (%)/mean (± SD)	Presence n (%)/mean (± SD)	p-value
**Block 1**			
**Age (in months)**	34.8 (±8.6)	36 (±6.8)	0.16&
**Race/skin color**			
White	99 (79.2%)	112 (78.3%)	0.86
Non-white	26 (20.8%)	31 (21.7%)	
**Gender**			
Male	58 (46.4%)	77 (53.8%)	0.22
Female	67 (53.3%)	66 (46.2%)	
**Block 2**			
**Maternal Education**			
Higher or above	24 (19.8%)	21 (15.7%)	0.26
High School	58 (47.9%)	78 (58.2%)	
Elementary school	39 (32.3%)	35 (26.1%)	
**Family Income**			
≤ 700	8 (7.3%)	7 (5.5%)	0.58
> 700	102 (92.7%)	120 (94.5)	
**Number of children**			
2 or more	67 (55.8%)	57 (42.9%)	0.04
1	53 (44.2%)	76 (57.1%)	
**Block 3**			
**Attends Daycare**			
Yes	53 (44.9%)	57 (42.9%)	0.74
No	65 (55.1%)	76 (57.1%)	
**Number of Dental Appointments**			
1	61 (94.5%)	63 (44.7%)	0.42
2 or more	62 (50.4%)	78 (55.3%)	
**Block 4**			
**Breastfeeding**			
Breastfeeds/has already breastfed	103 (94.5%)	106 (89.1%)	0.14
Never	6 (5.5%)	13 (10.9%)	
**Bottle use**			
Never/Rarely	37 (33.9%)	42 (35.6%)	0.79
Always	72 (66.1%)	76 (64.4%)	
**Finger Suction**			
Never/no longer	76 (73.1%)	77 (67.0%)	0.32
Uses/sleep	28 (26.9%)	38 (33.0%)	
**Pacifier Suction**			
Never used/has already used and stopped	33 (30.8%)	16 (13.9%)	<0.001
Always use it to sleep	74 (69.2%)	99 (86.1%)	

& T test.

**Table 2:** Crude and adjusted prevalence ratios for breastfeeding, bottle use, and pacifier use due to malocclusion in children. Porto Alegre, RS

**Variable**	**Crude PR**	**p-value**	**Adjusted PR**	**p-value**	**Adjusted PR**	**p-value**
**Block 1**						
**Age**	1.01 (0.99-1.02)	0.20	1.01 (0.98-1.04)	0.55		
**Race/skin color**						
White	1					
Non-white	1.03 (0.64-1.64)	0.92	1.00 (0.62-1.63)	0.99		
**Sex**						
Male	1					
Female	0.87 (0.70-1.09)	0.23	0.83 (0.54-1.28)	0.39		
**Block 2**						
**Maternal Education**						
Higher or above	1					
Elementary school	1.01 (0.68-1.50)	0.95	1.09 (0.73-1.640)	0.66	0.53 (0.22-1.26)	0.15
High School	1.23 (0.87-1.73)	0.24	1.28 (0.90-1.80)	0.165	1.34 (0.78-2.31)	0.29
**Family Income**						
≤ 700	1					
> 700	0.86 (0.50-1.50)	0.60	0.81 (0.48-1.38)	0.44	0.82 (0.39-1.73)	0.61
**Number of children**						
2 or more	0.78 (0.61-0.99)	0.04	0.78 (0.61-1.01)	0.05	0.98 (0.64-1.49)	0.91
1	1					
**Block 3**						
**Attends Daycare**						
Yes	0.96 (0.76-1.22)	0.74	0.94 (0.74-1.20)	0.62	1.46 (0.96-2.23)	0.08
No	1					
**Number of Dental** **Appointments**						
1	1					
2 or more	1.10 (0.87-1.38)	0.43	1.05 (0.83-1.34)	0.66	1.14 (0.72-1.82)	0.57
**Block 4**						
**Breastfeeding**						
Breastfeeds/has already breastfed	1					
Never	1.35 (0.97-1.88)	0.08	1.38 (0.96-1.97)	0.08	1.44 (1.00-2.08)	0.05
**Bottle use**						
Never/Rarely	1					
Always	0.97 (0.74-1.25)	0.79	0.99 (0.76-1.30)	0.95	0.96 (0.74-1.25)	0.78
**Finger Suction**						
Never/no longer	1					
Uses/sleep	1.14 (0.88-1.48)	0.31	1.07 (0.81-1.42)	0.61	1.06 (0.80-1.41)	0.69
**Pacifier Suction**						
Never used/has already used and stopped	1					
Always use it to sleep	1.75 (1.15-2.67)	0.01	1.69 (1.10-2.61)	0.02	1.81 (1.14-2.86)	0.01

## DISCUSSION

The main results of the present study show that the prevalence of malocclusion in early childhood is high, affecting 53.4% of this population. The most prevalent malocclusion was the anterior open bite, and it was also found that there was a higher prevalence of malocclusion in children who were never breastfed and who always used a pacifier to sleep. This is one of the first studies that present the association between sucking habits and malocclusion in the primary dentition of young children cared for by a PHC service.

Most of the studies related to this theme were carried out with populations of school children between 5 and 12 years old^(10,20)^. However, malocclusions have an early manifestation, which is still marked in the primary dentition. It is in this context that PHC becomes a reference because it is an adequate place to stimulate healthy practices related to oral health. This happens because it manages to cover the different stages of development, following from the gestational period until the establishment of daily practices of the child, such as the use of a pacifier. The role of oral health education in primary care is crucial, as this motivational guidance work generates effective results^(21)^.

There are programmatic actions that encourage children's access in the first years of life to the oral health service, favoring the participation of these families in the educational programs offered by the service. In the same territory as the SSC-GHC, a study was carried out with children up to 4 years old, which showed that 78% of children went to the dentist at least once in the first four years of life^(22)^. Thus, it is evident that it is possible to prevent and intercept malocclusion in its initial period, as the user accesses the health service early. It is important to consider that the multifactorial etiology of malocclusion includes genetic factors and numerous environmental factors, which, added together, contribute to the emergence of different types of malocclusion^(2,13)^.

Similar results regarding the higher prevalence of anterior open bite and its association with pacifier use were evaluated in a study also conducted in Brazil, in the southeastern region of the country^(23)^. This association was also confirmed by a systematic review of the literature^(24)^. Likewise, the literature proves the importance of breastfeeding in preventing anterior open bite in children with mixed dentition^(20)^. Breastfeeding is recommended during the first six months of the baby's life and is a protective factor for the imbalance of the stomatognathic system^(25)^. Children with shorter breastfeeding times have a higher prevalence of harmful oral habits and posterior crossbite in the mixed dentition^(10)^. The most common harmful habits are the use of a bottle (87.2%) and the use of a pacifier (39%), and these habits may be associated. Even with the presence of non-nutritive sucking practices, 78.9% of the population can still present normal occlusion after receiving exclusive breastfeeding for at least three months^(10)^.

There are few studies on this theme using a hierarchical model, with the age group of children under five years old and users of a Primary Health Care service. Previous studies have also used this model to identify factors associated with malocclusion^(26,27)^. However, the research that evaluated the dental characteristics and needs within the PHC did not define the age range for early childhood^(26)^. A study that used multilevel analysis in a population of preschoolers demonstrated the protective effect of breastfeeding from 9 to 12 months for malocclusion. Regardless of the time, breastfeeding is a protective factor for malocclusion and the presence of an anterior open bite^(28)^. Also, breastfeeding for at least six months has a protective effect against increased overjet, posterior crossbite, and crowding^(9)^.

Among the possible limitations for this study, there are those of cross-sectional studies because they do not allow establishing relationships of causes and effects, as well as changes over time. Another important consideration of this study is the borderline outcome (PR = 1.44; 95%CI 1.00-2.08) for breastfeeding. This result requires parsimony in its interpretation since the prevalence ratio is variable and with a lower limit awfully close to 1. It is expected that more studies will be conducted to confirm this finding.

## CONCLUSION

In conclusion, the prevalence of malocclusion in this population was high, and this condition was associated with behavioral habits, such as the use of a pacifier. Children who were never breastfed and used a pacifier to sleep had a higher prevalence of malocclusion, which shows that measures to prevent the incorporation of harmful oral habits should be encouraged early, preferably through a multidisciplinary health team with speech therapists, nurses, nutritionists, among others. Furthermore, intersectoral actions, to accompany mothers during pregnancy and child development, seeking to ensure children's oral health and, consequently, quality of life linked to oral health for parents and children.

When interpreting the results of this study, the importance of considering the generalization capacity of the sample is emphasized. Different regions may present variability in the results given the importance of socioeconomic, cultural, and genetic factors associated with the outcome.
